# Effects of pathogen infection and *Rhizobium* inoculation on instantaneous and long-term water use efficiency of peanut with and without drought

**DOI:** 10.3389/fmicb.2025.1612341

**Published:** 2025-06-24

**Authors:** Girmaye Benti Regassa, Yuxian Zhang, Yifan Shen, Liwei Zhang, Jiating Zhang, Yinzhan Liu, Guoyong Li, Rui Xiao, Zhongling Yang

**Affiliations:** ^1^International Joint Research Laboratory for Global Change Ecology, School of Life Sciences, Henan University, Kaifeng, Henan, China; ^2^Xiaoqinling Ecological Restoration Field Observation and Research Station of the Yellow River Basin, Henan University, Kaifeng, China

**Keywords:** peanuts, drought, greenhouse pot, pathogen, symbiosis, water use efficiency

## Abstract

**Introduction:**

Water Use Efficiency (WUE) is one of the critical indicators to characterize plant adaptation to arid environments, however, the effects of pathogens infection and *Rhizobium* symbiosis on WUE are not considered in contexts of water stress.

**Methods:**

A study was conducted in a greenhouse pot to examine the effects of changed soil water conditions on instantaneous Water Use Efficiency (WUE_i_) and long-term Water Use Efficiency (WUE_L_) under inoculation *Rhizobium*, inoculation *Fusarium* sp., and co-inoculation *Rhizobium* and *Fusarium* sp.

**Results:**

The results showed that inoculation *Fusarium* sp. and co-inoculation *Rhizobium* and *Fusarium* sp. reduced WUE_i_ by increasing net photosynthetic rate without drought. Inoculation *Fusarium* sp. and co-inoculation *Rhizobium* and *Fusarium* sp. reduced WUE_i_ by decreasing plant height with drought. Inoculation *Rhizobium* and *Fusarium* sp. significantly reduced WUE_L_ by lowering intercellular CO_2_ concentration without drought. Inoculation *Rhizobium* reduced WUE_L_ by increasing root nodule number with drought. In contrast, drought had no effect on either WUE_i_ or WUE_L_ without inoculation.

**Discussion:**

The results suggest that *Fusarium* sp. infection is detrimental to instantaneous Water Use Efficiency while inoculation *Rhizobium* is unfavorable to long-term Water Use Efficiency, regardless of drought effects. Our findings provide a new insight for developing effective water use strategies after pathogen infection or *Rhizobium* symbiosis under increased precipitation scenarios.

## 1 Introduction

Global change has intensified hydrological cycles, increasing the intensity and frequency of drought occurrences (Yang et al., 2020). Drought can inhibit plant growth and development, and even lead to plant mortality by lowing stomatal conductance and reducing photosynthetic rate (McDowell et al., 2013; Furlan et al., 2016). Therefore, it is of great significance to explore effective methods to improve plant water stress tolerance and drought resistance strategies (Mabrouk et al., 2022; Hidalgo-Hidalgo et al., 2022; Felipe et al., 2023).

Improving water use efficiency (WUE) is one of the crucial strategies for plant adaptation to arid environments (Domec et al., 2017). Plants employ various strategies to resist drought, including drought avoidance, drought tolerance, and drought resistance (Jewaria et al., 2021; Gupta et al., 2020). Under drought stress conditions, plants reduce evaporation loss by adjusting the size of their stomata, thereby slowing down the rate of water loss (Sun et al., 2020). They also enhance root growth to increase water uptake, improving tissue water status, and utilizing water resources more efficiently (Nguyen et al., 2022). Furthermore, plants adapt to arid environments by altering physiological characteristics, including chlorophyll content and leaf area (Lauri et al., 2016; Guzzo et al., 2021). Therefore, the mechanisms of plant to resist drought depend on plant morphology and physiological traits.

Pathogens can also alter WUE by affecting plant hydraulic properties and regulating stomatal behavior (Melotto et al., 2006; McElrone et al., 2008). Previous study indicates that pathogens significantly influence photosynthetic parameters in various ways (Gortari et al., 2018; Homet et al., 2019; Li et al., 2022; Murria et al., 2022). For example, the net photosynthesis rate in most plants decreases significantly after infection with pathogens, and this is observed in poplars infected with rust disease (Gortari et al., 2018) and pearl millet [*Pennisetum glaucum (L.) R. Br.*] infected with *Sclerospora graminicola* (Murria et al., 2022). In contrast, studies have reported an increase in the net photosynthesis rate of *Quercus suber* seedlings infected with Phytophthora *cinnamomi* (Homet et al., 2019) and oat leaves infected with Puccinia *graminis* f. sp. *avenae* (Li et al., 2022). Plants can also symbiosis with beneficial microorganisms, such as *Rhizobium* and *Arbusculia*, to improve plant drought resistance (Rolli et al., 2015). *Rhizobium* is a common plant growth promoting bacteria in pulses and other crops. *Rhizobium* fixes nitrogen in the nodules through symbiosis, supporting the metabolism of plant. Studies have shown that the selection of suitable and effective *Rhizobium* can improve the symbiotic combination of plants and *Rhizobium* under drought stress, improving the productivity of legumes (Kibido et al., 2020). Therefore, damage caused by water scarcity can be reduced by inoculating drought-tolerant *Rhizobium* strains (Barbosa et al., 2018). These findings suggest that complex physiological and biochemical regulatory mechanism works during the interaction of plants and microbes under drought conditions. However, the roles of pathogens infection and symbiosis with beneficial microorganisms in influencing WUE are often overlooked.

As one of the world’s most important oil and cash crops, peanut has significant industrial value and development potential. Elucidating the dynamics of peanut-microbe interactions and their regulating effects on water use efficiency are crucial for maintaining peanut yield. Here, we conducted a greenhouse pot experiment by inoculating peanuts with *Rhizobium* and *Fusarium* sp. during pod-setting stages (75 days after inoculation) to investigate how *Rhizobium* and pathogen infections affect peanut water use efficiency (WUE) both at leaf and whole-plant level under water stress conditions. Specifically, we hypothesize that: (1) water use efficiency at the leaf-level and/or whole-plant level will be enhanced under drought conditions, and inoculation *Rhizobium* may further promotes the positive effect of drought on water use efficiency by aiding peanuts to fix nitrogen; (2) infection by *Fusarium* sp. may reduce water use efficiency at the leaf-level and/or whole-plant level by infecting peanuts with diseases, and drought may exacerbate the negative effects of *Fusarium sp* inoculation on water use efficiency.

## 2 Materials and methods

### 2.1 Experimental site

This experiment was carried out in the experimental greenhouse of sustainable agricultural ecology innovation site (114° 18′ 17′′E, 34° 49′ 15′′ N) in Jinming Campus of Henan University. The area is located in the hinterland of North China Plain, which belongs to temperate monsoon climate with four distinct seasons. The average annual temperature is 14.52°C, the average annual precipitation is 627.5 mm, and the precipitation is mostly concentrated in July and August. The soil type used in this experiment is sandy soil, which is suitable for peanut growth.

### 2.2 Experimental materials

The peanut used in the experiment was the “Kainong 98” variety, which was jointly cultivated by Kaifeng Academy of Agricultural and Forestry Sciences and Henan University. The tested *Fusarium* sp. was provided by China Agricultural Microbial Strain Storage Management Center, and the strain number was ACCC 36194. *Brady Rhizobium hongdouense* (ACCC 14082) was provided by China Agricultural Microbiological Culture Collection and Management Center. The sandy soil of 0–30 cm layer in Kaifeng local farmland was used as the culture matrix, and the large soil blocks and plant residues were removed by 2 mm sieve. The gamma ray was used to sterilize soil in Piaohe Longxiang Radiation Technology Co., Ltd. The active microbial strain was not detected in the sterilized soil by the company’s quality inspection. The bottom of the PVC pipe with an inner diameter of 25 cm and a depth of 1 m is sealed as a flowerpot. After the two wire ropes are knotted and closed into a ring, they cross from the bottom and are fixed along the side wall. Two handles are formed on both sides of the flowerpot to facilitate the lifting and weighing of the small gantry crane.

### 2.3 Experimental design

The top of the experimental greenhouse is covered with a glass pane with good light transmittance, and a rain-blocking mesh cloth is set around to prevent rainwater from entering. The bottom of the greenhouse is a pool with a depth of 1 m. The edge of the pool is 2 m wide from the edge. The width of the pool is 2.4 m and the length is 13.4 m. The PVC flowerpot is neatly placed in the pool. A row of wooden boards is set up between each row of flowerpots to facilitate walking and shading the side wall of the flowerpot.This experiment included water supply (WW: 70% field capacity, natural water conditions; SD: 35% field capacity, water limitation group) and inoculation of microorganisms [no inoculation of microorganisms (recorded as C), inoculation of *Rhizobium* (recorded as R), inoculation of *Fusarium* sp. (recorded as X), and simultaneous inoculation of *Rhizobium* and *Fusarium* sp. (recorded as XR)], a total of 8 treatment combinations. Each treatment had 10 replicates, and totally of 80 plots. The field water capacity was measured by the determination method of soil specific gravity, 70% field capacity was watered every 3 days and 35% field capacity watered every 5 days to maintain a stable soil water content. The inoculation of rhizobia was completed during the seedling period, and the inoculation of *Fusarium* sp. was carried out during transplanting. After transplanting, the pots were divided into 10 blocks, and 8 treatments in each block were randomly placed. The harvest was carried out in the pod stage (75 days after inoculation with *Fusarium* sp.).

### 2.4 Preparation of *Rhizobium* and *Fusarium* sp.

The activated *Rhizobium* strains were inoculated into liquid medium [sucrose 10 g, K_2_HPO_4_ 0.5 g, MgSO_4_⋅7H_2_O 0.2 g, CaSO_4_ 0.2 g, NaCl 0.1 g, yeast powder 1 g, NaMoO_4_(1%) l mL, Iron citrate (1%) 1 mL, Boric acid (1%) 1 mL, MnSO_4_ (1%) 1 mL, H_2_O 1 L, pH (6.8–7.0)] at 28–30°C for 2.5 days to the logarithmic phase, 480 mL of the bacterial solution was centrifuged at 6,000 r/min for 10 min to collect the bacteria, and then re-suspended with 220 mL of 0.85% NaCl solution to obtain the bacterial suspension. The bacterial suspension was measured by plate colony counting method. The viable bacterial concentration was 7.2 × 10^9^ CFU/mL. Inoculation of *Fusarium* sp. into liquid medium (peel potato 200 g, cut into small water 1,000 ml boil 30 min block, filter to remove the potato block, the filtrate fill to 1,000 ml, add glucose 20 g, agar 15 g, dissolved after packing, 15 pounds sterilization 30 min) after 2.5 days of shaking culture at 25∼28°C to the logarithmic phase, the bacterial liquid was centrifuged at 8,000 r/min for 10 min to collect the bacteria. After the supernatant was removed, it was re-suspended with 500 mL water to obtain the bacterial suspension. The dry weight method was used to determine the concentration of *Fusarium* sp., and the measured concentration was 30.7 mg/mL.

### 2.5 Seedlings, transplanting, and harvests

Peanut seeds and plastic non-porous seedling box (upper diameter 10 cm, lower diameter 8 cm, height 9 cm) were soaked in 70% alcohol for 5 min and 1 h, respectively, and washed three times with sterile water. Distilled water was added to the sterilized soil, and the soil was wet with sterile gloves. Starting from June 5, the seedlings were boxed and inoculated with *Rhizobium*. After adding 510 g of soil in the seedling basin, two peanut seeds were placed, and then 2 mL of *Rhizobium* solution was added around the peanut seeds. Finally, 100 g of soil was evenly covered to the upper layer. The inoculation of *Fusarium* sp. was the same as above, but 6 mL *Rhizobium* solution was added around the peanut seeds. The treatments without inoculation of *Rhizobium* and *Fusarium* sp. were replaced with 2 mL0.85% NaCl solution and 6 mL distilled water, respectively. Before seedling emergence, the seedling box should be covered to avoid excessive temperature or direct sunlight leading to excessive evaporation of soil moisture. During the seedling period, the seedling box should be watered with a spray pot every morning and evening to keep the soil moist.The water content in the soil was measured in advance after the soil was sterilized, and then the sterilized soil and distilled water were added to the stirrer in proportion to stir evenly, so that the soil reached 35% field capacity (35% FC, recorded as SD) and 70% field capacity humidity (70% FC, recorded as WW). The soil was divided into PVC tubes, and the total weight of the tube and soil was recorded. Three peanut seeds with similar weight and good plumpness were selected and planted in the soil of PVC pipe. After the peanut germinated, the other two seedlings were removed to ensure that there was one peanut seedling in each PVC pipe. The weighing method was used to control the water of the pot every 3 days and the watering amount was recorded every time.

Five blocks were randomly selected to harvest plants at the flower needle and podding stage of peanuts, respectively. The peanuts in the PVC pipe were poured out together during harvest, and then the peanut roots were carefully removed from the soil to maintain the integrity of the roots as much as possible.

### 2.6 Determination of growth traits and WUE_L_

At the harvest time of the two periods, three individuals of each treatment were randomly selected, and each individual was separated from the junction of the above-ground and below-ground parts of the main stem, washed with tap water, and loaded into a marked envelope. The fresh weight of the leaves was immediately measured after collection to minimize the impact of water evaporation and record the number of nodules in the root (RNN). After measuring the fresh weight, the stems, leaves, pods, and roots of the peanut were dried at 65°C for 48 h before weighing the biomass of each part. Then the root-shoot biomass ratio of each plant was calculated. The concentration or presence of *Fusarium* sp. in peanut roots is assessed using the agar dilution plate method, which quantifies the number of colony-forming units per gram of dry root weight. Three early morning water potential and noon water potential of peanuts were measured. Pre-dawn water potential measurements were measured between 4:00 and 5:00, and midday water potential measurements were measured between 12:00 and 13:00. Measurements were performed using a pressure chamber instrument. Cut branches or leaves of plants were sealed into the pressure chamber of the device, and pressure was gradually increased until water begins to flow from the cut surface. The pressure at this point represents the water potential of the plant sample. Soil moisture was calculated by the following formula: Soil moisture (%) = [(Wet Weight - Dry Weight)/Dry Weight] × 100%.

### 2.7 Gas exchange parameters and WUE_i_

Water use efficiency (WUE) is an important parameter reflecting the water use characteristics of plants, which represents the assimilation amount produced by consuming unit water. Long-term water use efficiency (WUE_L_) was the ratio of plant biomass to total transpiration water during the experiment (Liu, 2012). Between 9:00–12:00 on sunny days, LI-6400 (Beijing Ligaotai Technology Co., Ltd.) was used to measure the net photosynthetic rate (Pn), transpiration rate (Tr), stomatal conductance (Gs), and intercellular CO_2_ concentration (Ci) of mature and intact plant leaves for each treatment, with three replicates for each measurement. Instantaneous water use efficiency (WUE_i_) was calculated as the ratio of the net photosynthetic rate (Pn) to the transpiration rate (Tr).

### 2.8 Data analysis

One-way variance analysis was used to test the effects of inoculation on peanut WUE_i_, WUE_L_, plant height, nodule number (RNN), net photosynthetic rate (Pn), transpiration rate (Tr), stomatal conductance (Gs) and intercellular carbon dioxide concentration (Ci) with drought and without drought in 2022, and inoculation was viewed as fixed factors. The Bonferroni *post-hoc* test was applied to examine significant differences among the treatments. One-way analysis of variance was also used to examine drought effect in the unvaccinated plots. Linear regression analysis was used to analyze the relationships of RNN, plant height, Pn, Tr, Gs, and Ci with WUE_i_ and WUE_L_. All statistical analyses were conducted using R software (R Core Team, 2023, version 4.2.3). Structural equation models (SEMs) were used to assess the effects of inoculation on WUE_i_ and WUE_L_ under both drought and non-drought conditions by examining changes in Pn, Gs, and Ci under non-drought conditions, and plant height, RNN, and Ci under drought conditions in 2022. Based on the potential relationship between peanut functional traits, gas exchange parameters, and WUE_i_ and WUE_L_, a prior model was established. The model fit was assessed using the chi-square test and the minimum Akaike Information Criterion (AIC). SEM analysis was performed using AMOS 24.0.

## 3 Results

### 3.1 Rhizobium and *Fusarium* sp. inoculation

**FIGURE 1:**
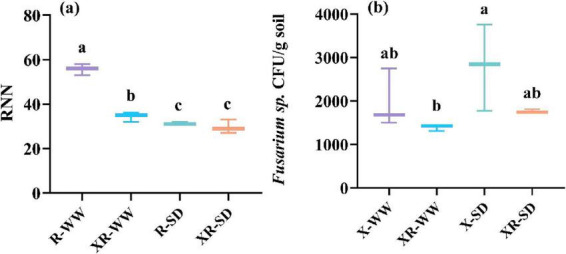
The number of nodules **(a)** and the concentration of *Fusarium* sp. CFU/g soil **(b)** after inoculated with *Rhizobium* (R) or *Fusarium* sp. (X) and simultaneous inoculated with the two microorganisms (XR) during the pod stage under SD and WW conditions. RNN: the number of *Rhizobium* nodules in peanut roots; *Fusarium* sp. CFU/g soil: the number of *Fusarium* sp. colony-forming units per gram of soil; WW: 70% field capacity, natural water conditions; SD: 35% field capacity, water limitation group. Different letters indicate significant differences at *p* < 0.05.

Nodules were only present in peanuts inoculated with *Rhizobium* (R) and co-inoculated with *Fusarium* sp. and *Rhizobium* (XR) under both 35 and 70% field capacity (Figure 1a). The average number of nodules for R- and XR-inoculated peanuts was 54.04 and 35.50, respectively, under 70% field capacity. The average number of nodules for R- and XR-inoculated peanuts was 31.47 and 30.30, respectively, under 35% field capacity (Figure 1a). *Fusarium* sp. was detected in peanuts inoculated with *Fusarium* sp. and co-inoculated with *Fusarium* sp. and *Rhizobium* (XR) in both 35 and 70% field capacity. The concentration of *Fusarium* sp. was 1976.34 and 1391.04 CFU/g in peanuts when inoculation with X and XR, respectively, under 70% field capacity. The concentrations were 2795.13 and 1764.79 CFU/g in peanuts when inoculation with X and XR, respectively, under 35% field capacity (Figure 1b).

### 3.2 Leaf water potential and soil water content

**FIGURE 2:**
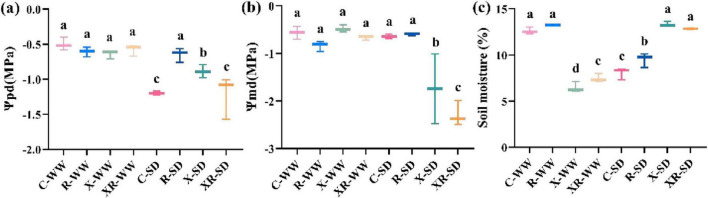
Ψ_pd_ **(a)**, Ψ_md_ **(b)**, and soil moisture **(c)** under uninoculated (C), inoculated with *Rhizobium* (R) or *Fusarium* sp. (X) and simultaneous inoculated with the two microorganisms (XR) at the pod stage under SD and WW conditions. Ψ_pd_: predawn water potential; Ψ_md_: midday water potential; WW: 70% field capacity, natural water conditions; SD: 35% field capacity, water limitation group. Different letters indicate significant differences at *p* < 0.05.

Pre-dawn water potential, midday water potential, and soil moisture were −0.50 MPa, −0.56 MPa, and 12.62%, respectively, under 70% field capacity. Drought conditions (35% field capacity) increased the pre-dawn water potential by 138.47% (*P* < 0.001) and decreased the field water content by 34.98% (*P* < 0.001; Figures 2a–c). Inoculation X and XR reduced the field water content by 47.33% and 39.94% (*P* < 0.001; Figure 2c), respectively. Inoculation R decreased the field water content by 22.95% (*P* < 0.001; Figure 2c), inoculation X and XR increased the pre-dawn water potential by 76.61% (*P* < 0.01) and 143.46% (*P* < 0.001; Figure 2a), respectively, under 35% field capacity. Neither drought nor inoculation affected the midday water potential (Supplementary Table S1).

### 3.3 Effects of inoculation and drought on peanut WUE

**FIGURE 3:**
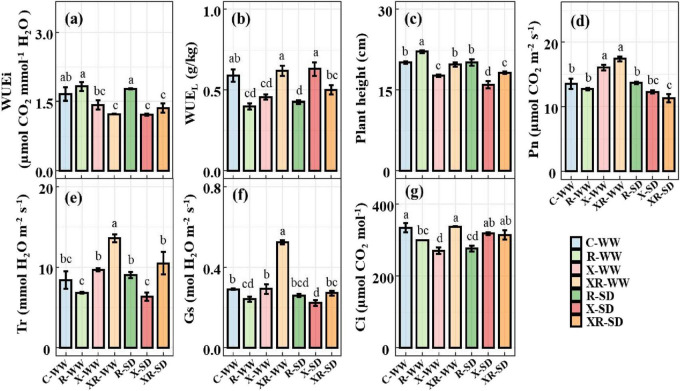
WUE_i_ **(a)**, WUE_L_ **(b)**, plant height **(c)**, Pn **(d)**, Tr **(e)**, Gs **(f)**, and Ci **(g)** of peanut without inoculation (C), inoculation Rhizobium (R) or *Fusarium* sp. (X), and simultaneous inoculation the two microorganisms (XR) at the pod stage under WW and SD, the values were mean ± standard error (*n = 3*). WW: 70% field capacity, natural water conditions; SD: 35% field capacity, water restriction group. Different letters indicate significant differences at *p* < 0.05.

WUE_i_ and WUE_L_ were 1.659 μmol CO_2_ mmol^–1^ H_2_O and 0.588 g⋅kg^–1^, respectively, under natural water conditions (70% field capacity). Drought had no effect on either WUE_i_ or WUE_L_ (Supplementary Table S1 and Supplementary Figure S1). Under natural water conditions, inoculation X and XR decreased WUE_i_ by 14.56% (*P* = 0.066) and 26.19% (*P* < 0.01, Figure 3a), respectively, while inoculation R did not affect WUE_i_; inoculation R and X significantly reduced WUE_L_ by 32.56% (*P* < 0.001) and 22.77% (*P* < 0.01), respectively, whereas inoculation XR did not affect WUE_L_ (Figure 3b). Under drought conditions, inoculation X and XR significantly reduced WUE_i_ by 27.31% (*P* < 0.01) and 18.26% (*P* < 0.05, Figure 3a), respectively; Inoculation R reduced WUE_L_ by 27.82% (*P* < 0.001), whereas inoculation XR did not affect WUE_L_ (Figure 3b).

### 3.4 Effects of inoculation and drought on peanut morphological and physiological traits

Drought decreased plant height (*P* < 0.05) and Pn (*P* < 0.01), but did not affect root nodule number, Tr, Gs, or Ci (Supplementary Table S2). Under natural water conditions, inoculation R increased plant height (*P* < 0.01), and decreased Gs and Ci (*P* < 0.05, Figures 3c,f,g); inoculation X increased Pn (*P* < 0.01, Figure 3d), and decreased plant height (*P* < 0.001, Figure 3c) and Ci (*P* < 0.001, Figure 3g); inoculation XR increased Pn, Tr, and Gs (*P* < 0.001, Figures 3d–f). Under drought conditions, inoculation R significantly decreased Ci (*P* < 0.001, Figure 3g); inoculation X significantly reduced plant height (*P* < 0.001) and Gs (*P* < 0.01, Figures 3c,f); inoculation XR significantly reduced plant height and Pn (*P* < 0.01 Figures 3c,d).

### 3.5 Factors affecting WUE_L_ and WUE_i_

**FIGURE 4:**
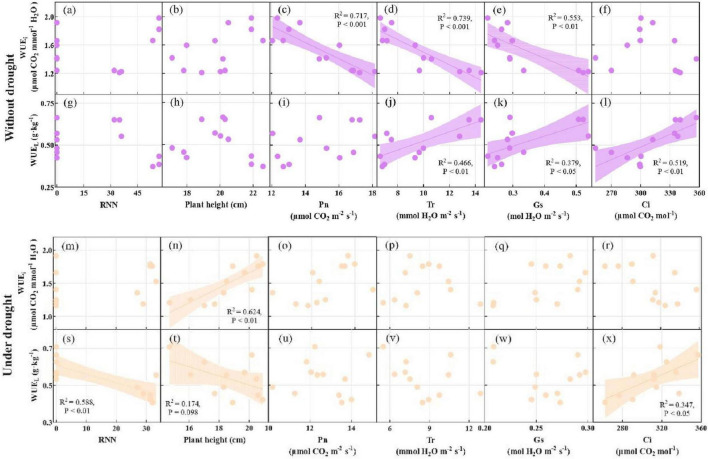
The linear correlation analysis of RNN, plant height, Pn, Tr, Gs, and Ci with WUE_i_ and WUE_L_ without drought **(a–l)** and under drought **(m–x)** at the pod stage. WUE_L_, long term water use efficiency; WUE_i_, instant water use efficiency; RNN, root nodule number; Pn, net photosynthetic rate; Tr, transpiration rate; Gs, stomatal conductivity; Ci, intercellular CO_2_ concentration.

Linear correlation showed that WUE_i_ was negatively correlated with Pn (*R*^2^ = 0.717, *p* < 0.001), Tr (*R*^2^ = 0.739, *p* < 0.001), and Gs (*R*^2^ = 0.553, *p* < 0.01), and WUE_L_ was positively correlated with Tr (*R*^2^ = 0.466, *p* < 0.01), Gs (*R*^2^ = 0.379, *p* < 0.05), and Ci (*R*^2^ = 0.519, *p* < 0.01) without drought. WUE_i_ was positively correlated with plant height (*R*^2^ = 0.624, *p* < 0.01), and WUE_L_ was positively correlated with Ci (*R*^2^ = 0.347, *p* < 0.05) and negatively correlated with root nodule number (*R*^2^ = 0.588, *p* < 0.01) and plant height (*R*^2^ = 0.174, *p* = 0.098) under drought (Figure 4).

**FIGURE 5:**
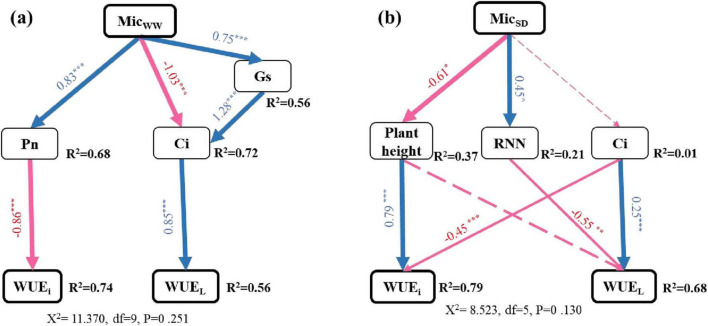
Structural equation models described the direct and indirect effects of inoculated with different microorganisms without drought **(a)** and under drought **(b)** on WUE_i_ and WUE_L_ of peanut at the pod stage. The arrow represents the direct influence determined by the path coefficient (i.e., the normalized factor load). R^2^ values represent the proportion of variance explained for each variable. The solid line arrows represent important relationships. The dotted arrow indicates an insignificant relationship. The blue arrow indicates a positive correlation, and the red arrow indicates a negative correlation. ***, **, and * represent significant level of 0.001, 0.01, and 0.05, respectively.

Under ambient water conditions, the SEM model showed that inoculation X and XR reduced WUE_i_ by increasing Pn, while inoculation R and X decreased WUE_L_ by lowering Ci (χ^2^ = 11.370, *P* = 0.251). Under drought, SEM model showed that inoculation X and XR reduced WUE_i_ by reducing plant height, and inoculation R reduced WUE_L_ by increasing the number of nodules (χ^2^ = 8.523, *P* = 0.130, Figure 5).

## 4 Discussion

### 4.1 Effect of inoculation on WUE_L_ and WUE_i_ under natural water condition

Inoculation *Fusarium* sp. and co-inoculation *Rhizobium* and *Fusarium* sp. decreased WUE_i_, and inoculation *Rhizobium*. and *Fusarium* sp. decreased WUE_L_. Pathogen infection reduced tomato development and diminished its water use efficiency (Buhtz et al., 2017). Pathogen infection (such as *Fusarium* sp.) may affect WUE mainly through two interrelated pathways. First, plants may regulate stomatal opening and closing (Huang et al., 2017), which directly affects gas exchange parameters (such as changes in stomatal conductance observed in this study), thereby affecting the calculation basis of WUE_i_. The enhancement of stomatal conductance may be an adaptation to the changes induced by inoculation, increasing carbon dioxide absorption. Therefore, it is essential to consider how external environmental factors (such as moisture, light, temperature, etc.) interact with the changes in inoculation and stomatal conductance, which may greatly influence the direction of the effect of inoculation on intercellular carbon dioxide concentration under different water conditions. In addition, plants might allocate more photosynthetic products (carbon resources) to defense responses, such as the synthesis of antimicrobial secondary metabolites (Kumari et al., 2024), and thereby redistribution resources away from growth and development. The transfer of this resource from growth and development (including potential water absorption and utilization efficiency optimization structure) is a key mechanism leading to long-term decline in WUE_L_ and WUE_i_ (as shown in the biomass change trend observed in this study). Although inoculation with *Rhizobium* does not increase WUE under natural water conditions, it is worth noting that the selection of suitable rhizobia strains may indeed improve water use (Del-Canto et al., 2023), especially under mild water stress (Bahador et al., 2023). However, nodule symbiosis itself requires resource input (such as maintaining nodules) and may change root structure and leaf physiology. The presence of rhizobia generally benefits plants, but their activity may inadvertently attract or increase plant susceptibility to certain pathogens and pests (Fahde et al., 2023; Liu et al., 2024), and thus affect the growth and biomass production. Following inoculation rhizobia, plants often undergo physiological adjustments to accommodate the new symbiotic relationship. These adjustments can include changes in root architecture and leaf area (Fahde et al., 2023). Although physiological changes are geared toward optimizing nitrogen fixation, they may simultaneously influence the overall accumulation of plant biomass. The benefits of promoting nitrogen fixation may not fully offset the cost of symbiosis or the potential consumption in resisting pathogens (such as co-inoculation), leading to neutral WUE.

### 4.2 Effects of inoculation on WUE_L_ and WUE_i_ under drought condition

WUE_i_ is decreased after inoculating with *Fusarium* sp. and co-inoculating with *Rhizobium* and *Fusarium* sp., and WUE_L_ is reduced after inoculation *Rhizobium* under drought conditions, which highlights the superimposed effect of drought and biotic stress. Drought itself forces plants to prioritize the basic metabolic functions required for survival at the expense of growth and efficiency (such as reduced WUE) (He et al., 2023). When superimposing pathogen infection, the situation is more severe. The damage of Fusarium to roots under drought (such as root structure damage and water transport disorder) directly limits the water absorption capacity, which is closely related to the observed decrease of WUE_i_. In order to cope with double stress of drought and Fusarium infection, plants need to mobilize more resources for osmotic regulation and synthesis of defensive secondary metabolites (such as flavonoids, phenols) (Khare et al., 2020; Ahmed et al., 2021; Khasin et al., 2021), causing the redistribution of carbon resources (from growth to defense and maintenance) and the decline in WUE_L_ (Condon, 2020).

Efficient water use allows plants to allocate more resources to support stem growth and expansion, particularly in environments where resources are scarce (Mundim and Pringle, 2018; Szczepaniec and Finke, 2019). However, both plant height and WUE decrease because energy and resources are reallocated to resist water stress in this study. In this study, inoculation of rhizobia under drought results in a decrease in WUE_L_, which is related to the cost of symbiotic nitrogen fixation in the absence of resources. In the period of reproductive growth, plants preferentially allocate resources to reproductive organs and nodules (Dolezal et al., 2021), and the effect is exacerbated by the limited resources under drought stress, resulting in a decrease in WUE_L_ after inoculation of rhizobia. The combined effects from prolonged drought and pathogen infection can induce significant physiological and metabolic challenges, further reduce photosynthetic efficiency and WUE. The destruction of root structure and function by drought reduces the ability of plants to absorb water and nutrients, which is also contributes to the decline in WUE_i_ (Farooq et al., 2019). Under severe drought, resources (especially water and carbon) are extremely limited (Zhang et al., 2021). Maintaining symbiotic nitrogen fixation and activating defense responses (rhizobia may also induce defense) consume a large amount of resources that can be used to maintain higher WUE_L_, resulting in a significant decrease in growth and WUE_L_. Consequently, a decline in WUE_i_ may mirror the decline in the plant’s photosynthetic capacity and water management efficacy. Moreover, the sustained reduction in leaf gas exchange parameters observed at podding stages underscores the irreversible harm on peanut leaves by severe drought (Zhang et al., 2021).

It should be noted that the experiment is carried out in a semi-open greenhouse environment (four sides ventilated, top glass covered). The measured data shows that the light intensity inside the greenhouse was about 80% of the natural field conditions, and the day and night temperature is significantly different from the field environment (temperature difference ≤ 1.5°C). Therefore, the semi-open greenhouse environment may amplify or weaken the observed drought effects by affecting plant transpiration rate, photosynthetic efficiency, and the intensity of stress response signals (Wang and Wang, 2023). For example, higher greenhouse temperature may accelerate soil water evaporation and plant transpiration, exacerbating the degree of water stress felt by plants (Zhang et al., 2022); specific light conditions may affect the ability of photosynthetic carbon assimilation, which in turn affects the total amount of resources that plants can use for defense and osmotic regulation (Heath et al., 2020). Therefore, it is necessary to consider the possible effects of specific light and temperature conditions in the greenhouse, and future studies consider the effects of greenhouses on light and temperature will be very valuable for accurately predicting the impact of drought.

## 5 Conclusion

This study explored the impact of rhizobia and *Fusarium* sp. inoculation, both individually and in combination, on leaf-level efficiency (WUE_L_) and instantaneous efficiency (WUE_i_) under different water conditions during the pod-filling stages. However, drought did not affect WUE_i_ and WUE_L_ without inoculation. In contrast, simultaneous inoculation *Rhizobium* and *Fusarium* sp. decreased WUE_i_ when inoculation of either *Rhizobium* or *Fusarium sp* alone. significantly reduced WUE_L_. Inoculation *Fusarium* sp. and co-inoculation *Rhizobium* and *Fusarium* sp. led to a decrease in WUE_i_, and inoculation *Rhizobium* decreased WUE_L_ under drought. The findings suggest that *Rhizobium* inoculation has crucial effects on long-term water use efficiency and *Fusarium* sp. inoculation largely regulates short-term water use efficiency. These insights are crucial for elucidating the dynamics of plant-microbe interactions and their regulating effects on water use efficiency under climate change scenarios.

## Data Availability

The original contributions presented in the study are included in the article/Supplementary material, further inquiries can be directed to the corresponding author.
