# Supplementary material for: Effects of pathogen infection and Rhizobium inoculation on instantaneous and long-term water use efficiency of peanut with and without drought
Source: Front Microbiol. 2025 Jun 24;16:1612341. doi: 10.3389/fmicb.2025.1612341 (PMC12235604; doi:10.3389/fmicb.2025.1612341)
Supplement: Supplementary file 1 [file Data_Sheet_1.docx]

**Fig. S1** WUE_i_ and WUE_L_ of uninoculated (C), inoculated with *Rhizobium* (R) or *Fusarium spp.* (X) and simultaneous inoculated with the two microorganisms (XR) at the pod stage under SD and WW conditions. Mean ± standard error (n = 12) was shown. WUE_L_, long term water use efficiency; WUE_i_, instant water use efficiency; WW: 70% field capacity, natural water conditions; SD: 35% field capacity, water restriction group.

Fig. S1


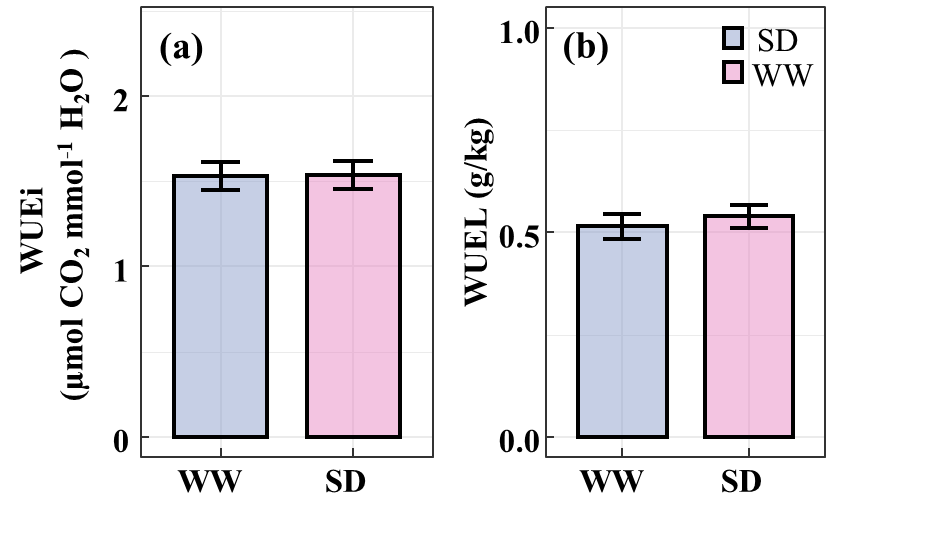


**Tab. S2** The significant effect of drought on each index at pod stage of uninoculated (C), inoculated with *Rhizobium* (R) or *Fusarium sp.* (X) and inoculated with these two microorganisms (XR) at the pod stage under SD and WW conditions were mean ± standard error (n = 12). WUE_L_, long term water use efficiency; WUE_i_, instant water use efficiency; Ψ_pd_, predawn water potential; Ψ_md_, midday water potential; USD, upper stomatal density; LSD, lower stomatal density; LDMC, leaf dry matter content; SLA, specific leaf area; WC, total watering amount; AGB, aboveground biomass; BGB, belowground biomass; RSR, root top ratio; RNN, root nodule number; Pn, net photosynthetic rate; Tr, transpiration rate; Gs, stomatal conductivity; Ci, intercellular CO_2_ concentration.

**Tab. S2**

| **Index** | **Degree of freedom** | **F** | **Significance** |  |
| --- | --- | --- | --- | --- |
| Ψ_pd_ | 1 | 155.394 | ＜0.001 |  |
| Ψ_md_ | | 1 | 0.931 | 0.389 |
| Soil moisture | 1 | 117.701 | ＜0.001 |  |
| USD | 1 | 0.757 | 0.394 |  |
| LSD | 1 | 5.345 | 0.031 |  |
| Vein density | 1 | 0.072 | 0.791 |  |
| WUE_L_ | 1 | 0.361 | 0.554 |  |
| LDMC | 1 | 0.005 | 0.944 |  |
| SLA | 1 | 1.051 | 0.316 |  |
| WC | 1 | 3.635 | 0.07 |  |
| AGB | 1 | 26.33 | 0 |  |
| BGB | 1 | 0.417 | 0.525 |  |
| Plant height | 1 | 7.624 | 0.011 |  |
| Root length | 1 | 14.735 | 0.001 |  |
| RSR | 1 | 27.211 | 0 |  |
| RNN | 1 | 0.713 | 0.407 |  |
| WUE_i_ | 1 | 0.002 | 0.964 |  |
| Pn | 1 | 7.88 | ＜0.01 |  |
| Tr | 1 | 1.073 | 0.312 |  |
| Gs | 1 | 2.905 | 0.102 |  |
| Ci | 1 | 0.452 | 0.508 |  |
